# Relationship between serum iron and the severity of hypertriglyceridemic acute pancreatitis

**DOI:** 10.1590/1806-9282.20250670

**Published:** 2026-04-20

**Authors:** Xuyao Yang, Lili Lan

**Affiliations:** 1 Chengdu Seventh People's Hospital, Department of Gastroenterology – Chengdu, China.

**Keywords:** Iron, Blood, Serum iron, Hypertriglyceridemia, Acute pancreatitis

## Abstract

**OBJECTIVE:**

The aim of this study was to investigate the relationship between serum iron and the severity of hypertriglyceridemic acute pancreatitis.

**METHODS:**

A total of 209 patients with newly diagnosed and treated hypertriglyceridemic acute pancreatitis admitted to our hospital from January 2021 to June 2024 were selected as the research subjects. The modified computed tomography severity index score was calculated to evaluate the severity of hypertriglyceridemic acute pancreatitis. According to the score values, they were divided into the mild group and the moderate-to-severe group. Another 60 healthy individuals during the same period were selected as the control group. The general clinical data of all enrolled subjects, as well as blood routine, high-sensitivity C-reactive protein, liver function, kidney function, random blood glucose, serum iron, serum calcium, serum triglyceride levels, modified computed tomography severity index scores, and abdominal computed tomography results, were collected. The differences in baseline data among the three groups were compared, and the correlations between different variables and modified computed tomography severity index scores were analyzed. Multiple linear regression analysis was used to further clarify the relationship between serum iron and modified computed tomography severity index scores.

**RESULTS:**

Among the baseline data comparisons among the three groups, except for hypertension, the differences in the remaining variables among the groups were statistically significant (p<0.05). Correlation analysis showed that blood routine, C-reactive protein, serum triglyceride, and random blood glucose levels were positively correlated with modified computed tomography severity index scores (r=0.601, r=0.492, r=0.604, r=0.432, all p<0.05), while serum iron was negatively correlated with modified computed tomography severity index scores (r=-0.475, p<0.05). The results of multiple linear regression analysis showed that in Model 1 and Model 2, serum iron was an independent influencing factor for modified computed tomography severity index scores (B=-0.084, B=-0.076, both p<0.05). In Model 3, after further adjusting for all remaining variables, serum iron was still an independent influencing factor for modified computed tomography severity index scores (B=-0.039, p<0.05), and the D-W value was the best, at 1.765.

**CONCLUSION:**

There is a certain negative correlation between serum iron and the severity of hypertriglyceridemic acute pancreatitis. That is, the lower the serum iron level, the higher the modified computed tomography severity index score, and the more severe the hypertriglyceridemic acute pancreatitis may be.

## INTRODUCTION

In recent years, with the improvement of people's living standards and the adjustment of dietary structure, the incidence of acute pancreatitis caused by hypertriglyceridemia (HTG-AP) has been increasing year by year. It has exceeded alcohol to become the second most common cause of acute pancreatitis^1^. Moreover, compared with acute pancreatitis caused by other reasons, HTG-AP is characterized by a younger age of onset, rapid progression, and a tendency to develop into severe cases, posing a serious threat to people's health^2^. Therefore, it is particularly important to pay attention to the risk factors that affect the early occurrence and development of HTG-AP.

Currently, in clinical practice, we have observed that HTG-AP patients often present with decreased serum iron (SI) levels upon early admission, along with abnormalities in amylase, blood calcium, and inflammatory markers. SI refers to the iron bound to transferrin in the plasma, which possesses immune-regulating functions and participates in the body's inflammatory response^3^. A significantly reduced SI level is a potential high-risk factor for poor prognosis or death when the body is under inflammatory attack or infection^4,5^. In the past, a few studies have analyzed the relationships between SI and acute pancreatitis. However, as HTG-AP becomes an increasingly common type of acute pancreatitis, its relationship with SI has not been reported. This paper aims to analyze the early serum iron levels in HTG-AP patients and explore their correlation with the severity of HTG-AP.

## PATIENTS AND METHODS

### Subjects

This cross-sectional study selected 209 patients with newly diagnosed hypertriglyceridemic acute pancreatitis (HTG-AP) admitted to our hospital from January 2021 to June 2024 as the study subjects, and another 60 healthy individuals during the same period were selected as the control group. Inclusion criteria were (1) newly diagnosed and admitted to the hospital within 2 weeks of onset; (2) diagnosed with HTG-AP according to the 2021 "Expert Consensus on the Diagnosis and Treatment of Hypertriglyceridemic Acute Pancreatitis in Emergency Departments"^2^, which first meets the diagnostic criteria for acute pancreatitis, followed by serum triglyceride (TG) levels reaching 11.3 mmol/L, or serum TG levels between 5.65 and 11.3 mmol/L with a chylous appearance, and exclusion of other causes of acute pancreatitis such as alcohol, biliary diseases, and so on. Exclusion criteria were (1) age <18 years; (2) pregnant or lactating women; (3) received or currently receiving iron supplementation within 3 months before admission; (4) blood system diseases; (5) acute or chronic hepatitis; (6) chronic blood loss due to various reasons (malignant tumors, peptic ulcers, menorrhagia, etc.); (7) acute exacerbation of chronic pancreatitis; (8) acute pancreatitis caused by other reasons (alcohol, biliary diseases, etc.). The study was approved by the hospital's medical ethics committee.

## METHODS

General information of all initially treated enrolled subjects was collected, including gender, age, body mass index (BMI), and history of hypertension, diabetes, and fatty liver disease. Laboratory data included white blood cell count (WBC), high-sensitivity C-reactive protein (CRP), alanine aminotransferase (ALT), aspartate aminotransferase (AST), albumin (ALB), blood urea nitrogen (BUN), creatinine (Cr), serum calcium (Ca), SI, serum triglyceride (TG), random blood glucose, abdominal CT results, and modified CT severity index (MCTSI) score.

The MCTSI score is the best scoring tool to evaluate the severity of HTG-AP^2^. It comprehensively assesses pancreatic inflammation, pancreatic necrosis, and extrapancreatic complications. Based on the severity of CT image morphology, it assigns scores ranging from 0 to 10, with higher scores indicating greater severity of HTG-AP. Specifically, scores of 0–2 are considered mild, 4–6 are moderate, and 8–10 are severe^6,7^_._

Statistical analysis was performed using SPSS 25.0 statistical software. Measurement data conforming to a normal distribution and homogeneity of variance were expressed as mean±-standard deviation ( $\bar{\text{x}}$ ±s), and one-way ANOVA was used for comparison between multiple groups. Measurement data with a non-normal distribution and unequal variances were expressed as M (P25~P75), and the Kruskal-Wallis H test was used for comparison between multiple groups. Categorical data were compared between groups using the χ² test. Spearman correlation analysis was used to analyze the correlation between SI and the severity of HTG-AP. Multiple linear regression analysis was used to analyze the relationship between SI and MCTSI. p<0.05 was considered statistically significant.

## RESULTS

### Comparison of baseline characteristics

This study included 209 patients with HTG-AP who were initially treated, with an average age of (41.59±10.24) years, and 60 healthy controls, with an average age of (47.13±9.51) years. Except for hypertension, the differences in other variables among the three groups were statistically significant (p<0.05) (Table 1).

**Table 1:** Comparison of variables among healthy controls and patients with different severities of hypertriglyceridemic acute pancreatitis.

Variable	Healthy (n=60)	Mild (n=137)	Moderate-severe (n=72)	Statistic	p
Age	47.13±9.51	41.74±10.21	41.29±10.35	F=7.068	0.001
Male	32 (53.5)	106 (77)	51 (70.8)	χ^2^=11.55	0.003
BMI	24.34±3.20	26.26±3.69	26.75±3.87	F=6.05	0.000
Hypertension	6 (10)	15 (10.9)	9 (12.5)	χ^2^=0.22	0.897
Diabetes	3 (5)	47 (34.3)	23 (31.9)	χ^2^=19.27	0.000
Fatty liver	8 (13.3)	85 (62.0)	50 (69.4)	χ^2^=50.23	0.000
WBC	5.2 (4.50–6.04)	13.07 (10.32–15.22)	13.92 (11.49–15.79)	H=131.12	0.000
CRP	0.95 (0.47–2.65)	12.03 (4.34–43.72)	12.18 (4.53–38.48)	H=96.97	0.000
ALT	21.45 (15–33)	32 (22–46.5)	31 (23–48.8)	H=14.67	0.001
AST	23 (19.13–28.9)	30 (24–37.4)	28.3 (20.13–47.03)	H=17.56	0.000
ALB	43.47±3.43	46.50±4.10	45.45±4.53	F=11.51	0.000
BUN	5.3 (4.7–6.3)	4.6 (3.7–5.8)	4.55 (3.7–5.2)	H=14.43	0.001
Cr	67.95 (56.58–82.85)	61 (45.5–74.5)	58 (40–74.25)	H=14.47	0.001
Ca	2.33 (2.28–2.41)	2.38 (2.3–2.47	2.33 (2.25–2.44)	H=8.21	0.016
SI	15.85 (11.35–21.93)	4.8 (2.85–7.95)	4.35 (3.2–7.28)	H=95.97	0.000
TG	1.44 (0.92–2.17)	16.92 (13.52–26.9)	19.97 (13.59–33.05)	H=140.08	0.000
Blood glucose	5.38 (4.6–6.28)	7.7 (5.8–12.64)	8.51 (6.5–13.38)	H=58.10	0.000

BMI: body mass index; WBC: white blood cell count; CRP: C-reactive protein; ALT: alanine aminotransferase; AST: aspartate aminotransferase; ALB: albumin; BUN: blood urea nitrogen; Cr: creatinine; Ca: serum calcium; SI: serum iron; TG: serum triglyceride.

### Correlation analysis of different variables with modified CT severity index

The results of Spearman correlation analysis showed that the levels of blood routine, CRP, TG, and random blood glucose were positively correlated with the MCTSI score (r=0.601, r=0.492, r=0.604, r=0.432, all p<0.05), and SI was negatively correlated with the MCTSI score (r=-0.475, p<0.05) (Table 2).

**Table 2:** Correlation analysis of different variables with modified CT severity index.

Variable	R	p
WBC	0.601	0.000
CRP	0.492	0.000
SI	-0.475	0.000
TG	0.604	0.000
Blood glucose	0.432	0.000

WBC: white blood cell count; CRP: C-reactive protein; SI: serum iron; TG: serum triglyceride.

### Multiple linear regression analysis

A regression model was established with MCTSI score as the dependent variable, gradually adjusting for independent variables such as age, gender, BMI, hypertension, and others to analyze the relationship between SI and MCTSI score. In Model 1 and Model 2, after adjusting for gender, age, BMI, hypertension, diabetes, and fatty liver, SI remained an independent influencing factor on MCTSI score (B=-0.084, B=-0.076, both p<0.05). In Model 3, after further adjusting for all remaining variables, SI continued to be an independent influencing factor on MCTSI score (B=-0.039, p<0.05), and the D-W value was optimal at 1.765 (Table 3).

**Table 3:** Multiple linear regression analysis of serum iron and modified CT severity index scores.

Variable	Model 1	Model 2	Model 3
SI	-0.084*	-0.076*	-0.039*
R	0.245	0.309	0.504
Adjusted ¢	0.233	0.291	0.471
F	21.360	16.692	15.031
P	0.000	0.000	0.000
D-W value	–	–	1.765

* p<0.000. Model 1 was adjusted for gender, age, and BMI. Model 2 was further adjusted for hypertension, diabetes, and fatty liver based on Model 1. Model 3 was further adjusted for WBC, CRP, ALT, AST, ALB, BUN, Cr, blood Ca, random blood glucose, and TG based on Model 2. SI: serum iron.

## DISCUSSION

Our findings reveal a novel association between SI levels and HTG-AP, suggesting that serum iron may play a significant role in the progression of HTG-AP severity. To our knowledge, few studies have yet explored this relationship. These results provide a new focus for future therapeutic strategies in HTG-AP.

HTG-AP, closely associated with significantly elevated serum triglyceride levels, has become a common type of acute pancreatitis^8^. In this study, we found that HTG-AP occurs more frequently in middle-aged males, which may be associated with a higher prevalence of metabolic disorders such as obesity and diabetes in this demographic compared to females^9^. A multicenter study from Taiwan also reported that the proportion of middle-aged males was significantly higher among patients with HTG-AP than in other etiology groups^10^. Spearman correlation analysis revealed a negative correlation between SI and MCTSI scores. Further multiple linear regression analysis, after adjusting for variables including gender, age, medical history, ALT, AST, BUN, Cr, serum calcium, and serum TG, identified SI as an independent influencing factor for MCTSI scores (B=-0.039, p<0.05). Patients with more severe HTG-AP exhibited lower SI levels. These findings underscore the importance of closely monitoring early SI changes in HTG-AP patients in clinical practice.

Early in the course of acute pancreatitis (AP), retrospective studies by Xie et al.^11^ and Xu et al.^12^ demonstrated that persistent hypoferremia is common among patients, and reduced SI levels were significantly associated with increased in-hospital mortality. Similarly, a pediatric study^13^ found that SI levels were markedly lower in children with severe AP compared to those with mild or moderate disease. The combination of SI with biomarkers such as procalcitonin, CRP, and D-dimer showed high predictive value for poor prognosis in pediatric AP. These findings are consistent with our results. However, a limitation of these earlier studies is that the AP population was not further stratified, and the relationship between SI levels and disease severity in HTG-AP remains unclear. Thus, our study aims to further investigate the association between SI and the severity of HTG-AP upon diagnosis.

Furthermore, a study by Deng et al.^14^ suggested that fluctuations in SI link ferroptosis to mortality and prognosis in acute pancreatitis (AP). Under critical conditions, cellular rupture releases iron ions, elevating SI levels and thereby inducing further ferroptosis, which exacerbates disease severity and mortality risk in AP. This offers an alternative perspective on the relationship between SI and AP. However, in our study, such an association was not observed. The discrepancy may be attributed to differences in the observed timepoints of disease progression and the focus between the two studies.

Currently, the mechanism underlying the correlation between SI and the severities of HTG-AP remains unclear. In the body's physiological metabolic reactions, SI plays a crucial role as a cofactor^15^. Relevant studies have pointed out that the imbalance of dynamic intracellular and extracellular transport of SI, leading to intracellular iron overload, can promote the increase of reactive oxygen species, cause damage and death of pancreatic acinar cells, and result in the gradual progression of acute pancreatitis^16^. In animal experimental models, iron overload accompanied by tissue cell damage and death has also been observed in the pancreatic tissue of mice with acute pancreatitis^17^. Therefore, it is believed that the decrease in SI during the occurrence and development of HTG-AP may be related to iron overload caused by the imbalance of iron transport in pancreatic tissue cells. On the other hand, the onset of HTG-AP can be accompanied by an inflammatory response, which can alter the distribution of iron in the body. Studies by JuOae Chang and Vela D^18,19^ have found that in an inflammatory state, the expression of iron exporter proteins in hepatocytes can decrease, and the expression of ferritin regulatory hormones can increase, inhibiting iron absorption from intestinal cells and release from macrophages, thus leading to a decrease in SI levels. Combined with the previous description, the decrease in SI levels further promotes the progression of acute pancreatitis.

This study has several limitations. Its single-center, cross-sectional design and relatively small sample size preclude the establishment of a causal relationship between SI and the severity of HTG-AP. Future prospective, multicenter studies with larger sample sizes are warranted. Further research is also needed to determine the optimal timing, dosage, and duration of iron supplementation, as well as to evaluate its effect on ameliorating disease severity in this patient population.

## CONCLUSION

This study further revealed a negative correlation between SI levels and the severity of HTG-AP at initial diagnosis. SI appears to play a significant role in the progression of HTG-AP. These findings suggest that, in clinical practice, close monitoring of SI levels upon diagnosis and during disease progression is essential. Timely correction of low SI may represent a critical measure to prevent the progression of HTG-AP to severe forms.

## Data Availability

The datasets generated and/or analyzed during the current study are available from the corresponding author upon reasonable request.
